# Low-Grade Inflammatory Mediators and Metalloproteinases Yield Synchronous and Delayed Responses to Mechanical Joint Loading

**DOI:** 10.1177/19476035231193089

**Published:** 2023-08-24

**Authors:** Conner W. Hutcherson, Michelle Mao, Bhaskar Thakur, Yasin Y. Dhaher

**Affiliations:** 1 Department of Orthopaedic Surgery, The University of Texas Southwestern Medical Center, Dallas, TX, USA; 2 Department of Physical Medicine and Rehabilitation, The University of Texas Southwestern Medical Center, Dallas, TX, USA; 3 Department of Population and Data sciences, The University of Texas Southwestern Medical Center, Dallas, TX, USA

**Keywords:** stress injury, articular cartilage, low-grade inflammation, serum biomarkers, osteoarthritis

## Abstract

**Objective:**

Mechanical loading is an essential factor for the maintenance of joint inflammatory homeostasis and the sensitive catabolic-anabolic signaling cascade involved in maintaining cartilage tissue health. However, abnormal mechanical loading of the joint structural tissues can propagate joint metabolic dysfunction in the form of low-grade inflammation. To date, few studies have attempted to delineate the early cascade responsible for the initiation and perpetuation of stress-mediated inflammation and cartilage breakdown in human joints.

**Design:**

Fifteen healthy human male participants performed a walking paradigm on a cross-tilting treadmill platform. Blood samples were collected before exercise, after 30 minutes of flat walking, after 30 minutes of tilted walking, and after an hour of rest. Serum concentrations of the following biomarkers were measured: interleukin (IL)-1β, IL-6, IL-10, tumor necrosis factor alpha (TNF)-α, matrix metalloproteinase (MMP)-1, MMP-3, MMP-9, MMP-13, transforming growth factor beta (TGF)-β, tissue inhibitor of matrix metalloproteinase 1 (TIMP)-1, and cartilage oligomeric protein (COMP).

**Results:**

Luminex Multiplex analysis of serum showed increased concentrations of COMP, IL-1β, TNF-α, IL-10, and TGF-β from samples collected after flat and cross-tilted treadmill walking compared to baseline. Serum concentrations of MMP-1 and MMP-13 also increased, but primarily in samples collected after tilted walking. Pearson’s correlation analysis showed positive correlations between the expression of COMP, TNF-α, IL-10, and MMP-13 at each study timepoint.

**Conclusion:**

Stress-mediated increases in serum COMP during exercise are associated with acute changes in pro and anti-inflammatory molecular activity and subsequent changes in molecules linked to joint tissue remodeling and repair.

## Introduction

Due to the avascular and aneural nature of the tissue structure, joint articular cartilage relies primarily on mechanosensitive and paracrine mechanisms to maintain its local environment. Chondrocytes, the sole cell type in joint articular cartilage, interpret loading on the surrounding extra cellular matrix (ECM) through mechanosensitive mechanisms that modulate the production of structural proteins, such as collagen and aggrecan, along with inflammatory and catabolic molecules.^1,2^ Under normal loading conditions, chondrocytes are able to maintain local tissue homeostasis; however, the presence of abnormal joint mechanics or overloading can initiate a metabolic imbalance stemming from the breakdown and release of structural ECM components.^3,4^ These matrix components are often classified as damage associated molecular patterns (DAMPs), indicating a damaged ECM, in which a tissue repair process begins to remove damaged structures before the formation of new ones.^5^ One well-established DAMP linked to cartilage damage, and is easily detectable in synovial fluid and serum, is cartilage oligomeric matrix protein (COMP).^6^ COMP is an ECM glycoprotein that is primarily characterized in cartilage-specific tissues.^7^ It plays an important role in stabilizing the interactions of ECM proteins, such as collagens I and II, which enhances the tensile mechanical strength of cartilage tissue.^8^ However, the presence of free COMP and DAMPs has been shown to bind and activate both integrin and toll-like receptor (TLR) families, which are present in the cellular membranes of chondrocytes, synovial fibroblasts, and macrophages.^9^ Following stimulation, these receptors initiate signaling of multiple intercellular pathways that result in the downstream production of pro and anti-inflammatory molecules associated with wound healing such as tumor necrosis factor (TNF)-α, interleukin (IL)-1β, IL-6, IL-10, and transforming growth factor beta (TGF)-β.^10^ The increased expression of these inflammatory mediators within the joint can create an imbalance of the tightly regulated anabolic-catabolic signaling axis, triggering the increased synthesis of enzymes from the group of metalloproteinases (MMPs), mainly collagenase 1 (MMP-1), stromelysin I (MMP-3), gelatinase A (MMP-9) and collagenase 3 (MMP-13), which further compromises various structural components of cartilage.^11,12^

Multiple *in vitro* experimental examinations have been conducted focusing on mapping the specific molecular pathways associated with synovial inflammation and cartilage destruction; however, the paradigms used in these controlled lab experiments naturally do not account for the *in vivo* biological complexity of the synovial and cartilage microenvironment. Stress-mediated inflammation in the synovium is generally considered a complex time-based process, in which the response (inflammation/tissue breakdown) exhibits unique features with respect to time after an applied stimulus (mechanical loading). Ideally, an *in vivo* experimental construct in humans should be employed to properly analyze the inflammatory molecular *milieu* of the joint using synovial fluid and synovial tissue (since they are taken directly from the affected site); however, the collection of these biological samples requires invasive procedures making it less than ideal for routine testing.^13^ In this context, the measurement of biomarkers in blood (serum or plasma) has been used as an alternative approach primarily due to the ease of sample acquisition.^14^ Although blood biomarkers have been examined in several joint specific studies involving strenuous exercise over a long period of time,^15-17^ these results do not capture the immediate response of key proteins over a short period of time and are often influenced by a wide range of confounding factors such as muscle damage and altered joint metabolism.^18^ For this reason, we incorporated a cross-tilting walking exercise paradigm to expose human lower body joint articular cartilage to a controlled magnitude of mechanical stimulus, as a means of initiating a synovial specific inflammatory response in the absence of any likelihood of exercise induced muscle damage.

The objective of this study was to indirectly analyze the signature of change of serum inflammatory and degradative molecular indicators under a controlled acute stress environment applied to the lower body joints of healthy human participants. Specifically, we hypothesized that (1) serum expression of COMP would increase as a result of an applied mechanical stimulus to the lower body joints, and that (2), a simultaneous and sequential inflammatory response, driven by the increased expression of pro- and anti-inflammatory cytokines as well as the degradative *metalloproteinase* will be positively associated with the expression of COMP. Identifying the differential effect of abnormal mechanical loading on lower limb joint inflammation would allow for the development of precise pharmacological interrogations of specific mechanosensitive molecular mechanisms and pathways associated with the pathogenesis of low-grade joint inflammation and the ensuing breakdown of articular cartilage.

## Methods

### Study Subjects

Fifteen healthy male subjects were recruited and enrolled in this study. All subjects were reported to be moderately active (less than 7 h of vigorous physical activity per week) and able to walk continuously for 1 hour without stopping. Subjects were instructed to abstain from any form of strenuous activity for at least 24 hours before the experimental session and to limit their normal dietary intake besides water in the 2 to 3 hours prior to the study. Subjects did not take any medication with anti-inflammatory actions for at least 1 month before the study. At the time of consent, a point of care anemia test was performed to ensure that participation in the study would not pose any risk to their wellbeing. All subjects were informed about the nature of the study and the associated risks and benefits, and appropriate consent was obtained pursuant to law. The study was approved by the UT Southwestern Institutional Review Board, and procedures were in accordance with the Helsinki declaration.

### Walking Exercise Procedure

**Figure 1.**
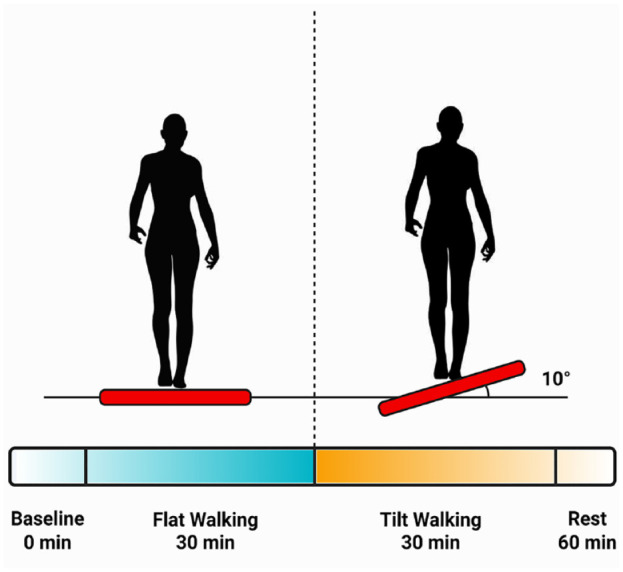
Conceptualization of the cross-tilting exercise paradigm. Blood samples were collected continuously at baseline (*t* = 0 min), at the end of flat walking (*t* = 30 min), at the end of tilted walking (*t* = 60 min), and after 60 minutes of rest (*t* = 120 min). Figure created with BioRender.com.

On arrival to the experiment site, subjects sat in a rested state for 30 min. During this phase, blood pressure and vitals were monitored and recorded. A baseline blood sample was collected by a certified nurse phlebotomist through a peripheral venous catheter inserted into the healthy subject’s peripheral vein. All whole blood samples were collected in gold top serum separator tubes (BD, Franklin Lakes, NJ) throughout the duration of the study. After the collection of the first sample, subjects were fitted into an overhead low friction safety harness system and began the walking procedure on the treadmill. The walking procedure consisted of 3 phases: a priming phase of 30 minutes of baseline flat walking on the treadmill; a stimulus phase of 30 minutes of cross-tilted treadmill walking (10° of tilt); and an hour of rest post exercise. Our choice of the 10° of tilt was informed by prior biomechanical studies using a lateral wedge insole design over ground walking.^19^ These studies indicated that at half of the present tilting angle (5°), significant changes in out-of-plane moments acting on the lower limb (specifically the knee) were observed. Blood samples were collected at the end of each walking phase and at the end of the post exercise rest period. The study design is illustrated in **Figure 1**. It is important to note that the transition from the flat to tilted configuration and back to flat was continuous, and the subject continued walking throughout the different phases.

### Whole Blood Processing and Storage

All collected whole blood was allowed to clot by leaving the collection tube undisturbed at room temperature for 15 to 30 minutes. The blood tubes were centrifuged at 1,900*g* for 12 minutes at 4°; then differentiated serum was immediately transferred into a clean polypropylene tube using a Pasteur pipette. The extracted serum was stored at −80°C in 100 µl aliquots. Batched analysis was performed when all samples on all participants were completed to avoid batch-dependent differences. The total amount of blood drawn was approximately 20 mL (3.25 teaspoon) per session and did not exceed 5 teaspoons. Blood samples that were hemolyzed, icteric, or lipemic were not included in the final analysis.

### Synovial and Muscle Damage Biomarker Assays

We investigated a range of serum biomarkers summarized in **Table 1**. On the day the assays were to be performed, all individual serum samples were transferred for analysis at the UTSW Genomics and Microarray Core Facility. All samples were analyzed in duplicate, and protein expression was measured via Luminex Multiplex Assay techniques using a standard curve run alongside the samples, as described previously.^20^ Biomarker concentrations measured below the lower limit of quantification were not included in the final analysis. Final marker calculations were made for each draw time point by averaging duplicate values and then normalized to baseline concentrations to control for varying baseline values between subjects.

**Table 1.** Summary of Biomarkers and Assays.

Biomarker	Details	Assay (Reference)	Supplier
COMP	Cartilage Matrix Protein	Luminex Multiplex Assay^20^	ThermoFischer, Waltham, MA
IL-1β	Inflammatory Cytokine	Luminex Multiplex Assay^20^	ThermoFischer, Waltham, MA
TNF-α	Inflammatory Cytokine	Luminex Multiplex Assay^20^	ThermoFischer, Waltham, MA
IL-6	Inflammatory Cytokine	Luminex Multiplex Assay^20^	ThermoFischer, Waltham, MA
IL-10	Inflammatory Cytokine	Luminex Multiplex Assay^20^	ThermoFischer, Waltham, MA
LAP (TGF-β)	Growth Factor	Luminex Multiplex Assay^20^	ThermoFischer, Waltham, MA
MMP-1	Collagenase I	Luminex Multiplex Assay^20^	ThermoFischer, Waltham, MA
MMP-3	Stromelysin I	Luminex Multiplex Assay^20^	ThermoFischer, Waltham, MA
MMP-9	Gelatinase B	Luminex Multiplex Assay^20^	ThermoFischer, Waltham, MA
MMP-13	Collagenase III	Luminex Multiplex Assay^20^	ThermoFischer, Waltham, MA
TIMP-1	MMP Inhibitor	Luminex Multiplex Assay^20^	ThermoFischer, Waltham, MA
CRP	Muscle Damage Marker	Vitros 350^21^	Ortho Clinical Diagnostics, Raritan, NJ
CK	Muscle Damage Marker	Vitros 350^21^	Ortho Clinical Diagnostics, Raritan, NJ
Myoglobin	Muscle Damage Marker	Sandwich ELISA^22^	Abcam, Waltham, MA

COMP = cartilage oligomeric protein; IL = interleukin; MMP = matrix metalloproteinase; TIMP-1 = tissue inhibitor of matrix metalloproteinase 1; CRP = C-reactive protein; CK = creatine kinase.

Serum levels of the muscle damage markers creatine kinase (CK) and C-reactive protein (CRP) were analyzed by the UTSW Metabolic Phenotyping Core using a Vitros 350^®^ clinical chemistry analyzer (Ortho Clinical Diagnostics, Raritan, NJ).^21^ Myoglobin levels in serum were determined using a sandwich ELISA (abcam Cat# ab171580, Waltham, MA) approach developed previously.^22^

### Statistical Analyses

All synovial biomarkers were assessed for normality assumption and found skewed. These skewed biomarker concentrations were transformed on a log scale first and summary statistics were generated with mean and standard deviation (SD). (See Suppl. Table S3) Graphical illustrations with mean and standard error (SEM) were also created for each biomarker. Pearson correlation coefficients were assessed for each pair of biomarkers at each time point and the correlation coefficients with *P* values were reported.

A linear, random intercept mixed effects model was utilized to assess the changes over time (within session) in the biomarkers of healthy male subjects. A generalized description of the structure and the utility of mixed effects modeling in biological applications have been described previously.^23^ All the analysis were carried out using standard statistical and data science software Stata 17.0 MP—Parallel Edition (StataCorp, LLC, College Station, TX) and GraphPad Prism version 9.4.0 (GraphPad Software, San Diego, CA)

## Results

### Participant Characteristics

Eighteen healthy male subjects completed the study screening. Three persons failed to fulfill the inclusion criteria. Fifteen were randomized. All randomized subjects completed the entirety of the exercise paradigm with no reported adverse events. Three subjects had partial data sets recorded due to complications with the IV catheter blood draw during exercise. An overview of the study population is displayed in **Table 2**.

**Table 2.** Demographics of Male Participants.

*N* = 15	Mean	SD	SEM
Age (years)	26.33	3.70	0.95
Height (cm)	175.90	6.87	1.78
Weight (kg)	81.05	20.53	5.30
BMI (kg/m^2^)	25.76	5.53	1.43

SEM = standard error of the mean; BMI = body mass index.

### Serum Biomarker Changes in Response to Mechanical Stimulus

**Figure 2.**
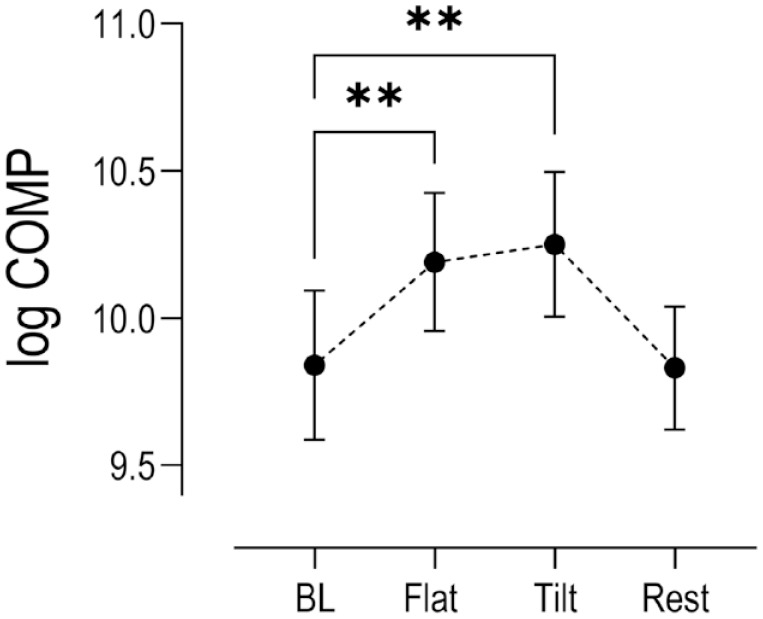
Changes in log serum COMP concentrations (*n* = 15) relative to baseline levels (BL) at key time points within the exercise paradigm. (Mean ± SEM). Stars indicate statistical significance (**P < 0.001) compared to baseline. COMP = cartilage oligomeric protein; SEM = standard error of the mean.

**Figure 3.**
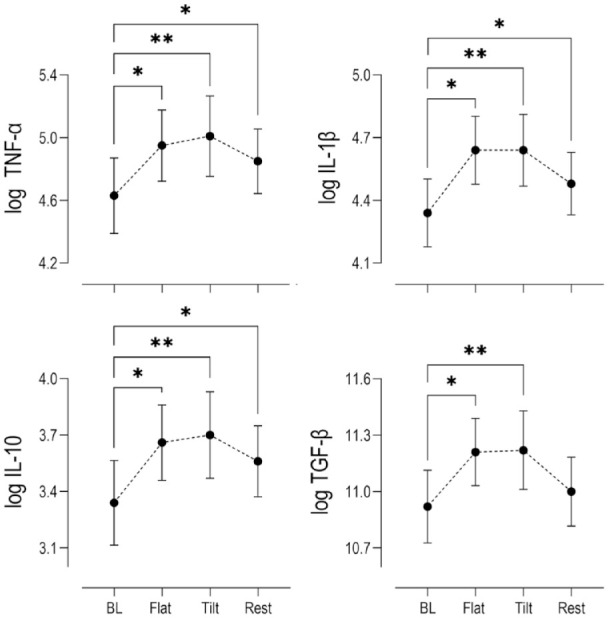
Changes in log serum concentrations (*n* = 15) relative to baseline levels (BL) of pro-inflammatory cytokines (TNF-α, IL-1β), anti-inflammatory cytokine (IL-10), and anabolic growth factor (TGF-β) at key time points within the exercise paradigm. (Mean ± SEM) Stars indicate statistical (*P < 0.05, **P < 0.001) compared to baseline. TNF-α = tumor necrosis factor alpha; IL = interleukin; TGF-β = transforming growth factor beta; SEM = standard error of the mean.

Log transformed serum biomarker concentrations calculated from samples collected at the end of 30-minutes flat walking period, at the end of 30-minutes tilted walking period, and after an hour of rest post exercise were compared to concentrations at rest prior to exercise with *P* < 0.05 set for statistical significance. Statistically significant increases in the mean expression of COMP were reported after 30 minutes of pre exercise flat walking and 30 minutes of subsequent tilted walking exercise (*P* < 0.001), followed by a return to basal levels after 1 hour of rest post exercise (**Fig. 2**). Significant increases in serum inflammatory biomarkers TNF-α (*P* = 0.002), IL-1β (*P* = 0.003), IL-10 (*P* = 0.003), and TGF-β (*P* = 0.013) were observed after 30 minutes of flat walking, and strong significant increases were detected after 30 minutes of subsequent tilted walking (*P* < 0.001) compared to baseline mean concentrations. However, after 1 hour of rest post exercise, while serum COMP returned to mean basal levels, concentrations of IL-1β (*P* = 0.05), TNF-α (*P* = 0.003) and IL-10 (*P* = 0.001) remained elevated. The relative mean changes for the present inflammatory biomarkers are summarized qualitatively in **Figure 3** and quantitatively in Suppl. Table S1.

**Figure 4.**
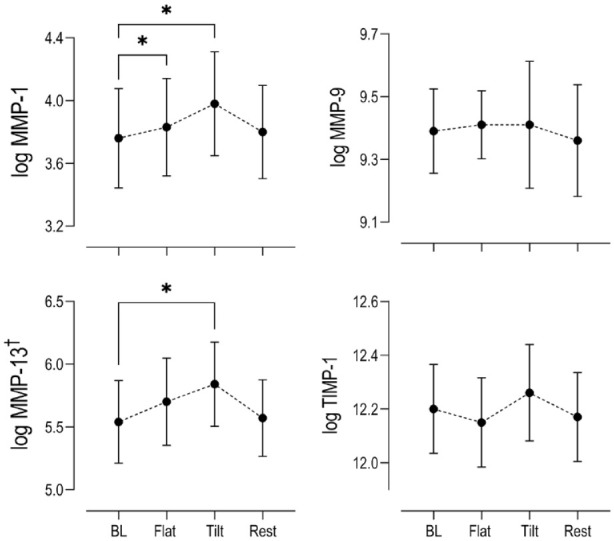
Changes in log serum concentrations (*n* = 15) relative to baseline levels (BL) of catabolic MMPs 1, 9, and 13 and inhibitor of MMPs (TIMP-1) at key time points within the exercise paradigm. (Mean ± SEM) Stars indicate statistical significance (*P < 0.05) compared to baseline. MMP = matrix metalloproteinase; TIMP-1 = tissue inhibitor of matrix metalloproteinase 1; SEM = standard error of the mean. ^†^:MMP-13 concentrations from 4 subjects were below Luminex assay detection threshold and not included in the final analysis (*n* = 11).

When comparing the mean serum MMP concentrations during exercise, only the concentrations for the collagenases MMP-1 and MMP-13 displayed any noticeable changes compared to baseline (**Fig. 4**). While serum MMP-1 levels displayed significant increases after 30 minutes of flat walking (*P* = 0.003) and 30 minutes of tilted walking (*P* = 0.010), MMP-13 concentrations were only significantly elevated after tilted walking (*P* = 0.002). Interestingly, both enzymes then promptly returned to baseline levels 1 hour post exercise. The mean changes in MMP-9 and tissue inhibitor of matrix metalloproteinase 1 (TIMP-1) were not statistically different from basal levels during or post exercise. All measured serum concentrations for MMP-3 and the cytokine IL-6 fell below the lower limit of the assay quantification across the cohort and were not included in the final analysis.

### Serum Biomarker Expression Correlations

**Figure 5.**
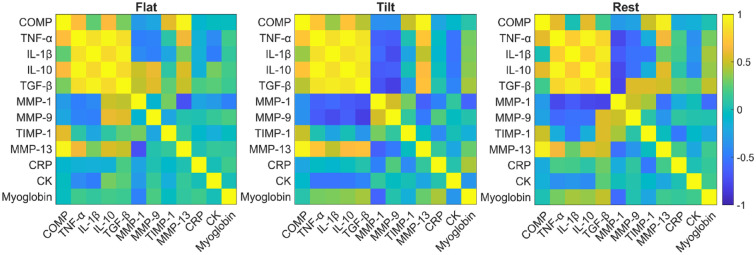
Pearson correlation coefficients were assessed and visualized as a heatmap for each pair of biomarkers detected from blood serum samples taken throughout the study. The color scheme with yellow and blue represents positive and negative correlations, respectively. *P* < 0.05 for r values greater than 0.51 and less than −0.51. COMP = cartilage oligomeric protein; TNF-α = tumor necrosis factor alpha; IL = interleukin; MMP = matrix metalloproteinase; TIMP-1 = tissue inhibitor of matrix metalloproteinase 1; CRP = C-reactive protein; CK = creatine kinase.

Throughout the duration of the paradigm, the temporal expression of COMP was strongly correlated with the expressions of TNF-α (Flat: *r* = 0.687, *P* = 0.010; Tilt: *r* = 0.731, *P* = 0.003; Rest: *r* = 0.583, *P* = 0.023), IL-10 (Flat: *r* = 0.669, *P* = 0.013; Tilt: *r* = 0.716, *P* = 0.004; Rest: *r* = 0.576, *P* = 0.025), and MMP-13 (Flat: *r* = 0.964, *P* < 0.001; Tilt: *r* = 0.968, *P* < 0.001; Rest: *r* = 0.935, *P* = <0.001). The expression of the biomarkers TNF-α, IL-1β, IL-10, and TGF-β were all positively correlated throughout each phase of the study (*P* < 0.001). The expression of MMP-13 was positively correlated to changes in TNF-α (*r* = 0.727, *P* = 0.017), IL-10 (*r* = 0.676, *P* = 0.032), and TGF-β (*r* = 0.696, *P* = 0.026) from samples collected after 30 minutes of tilted walking, but not after 30 minutes of flat walking. All serum correlation results are summarized in a heatmap format in **Figure 5**.

### Changes in Muscle Damage Biomarkers

**Figure 6.**
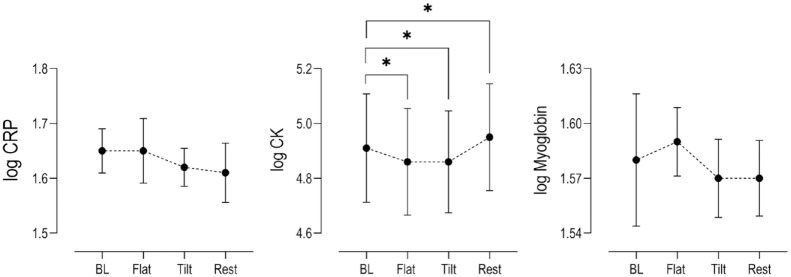
Changes in log serum concentrations (*n* = 15) relative to baseline levels (BL) of muscle damage biomarkers CRP, CK, and Myoglobin at key time points within the exercise paradigm (Mean ± SEM). Stars indicate statistical significance (*P* < 0.05) compared to baseline. CK concentrations from 1 subject was below the Vitros 350 assay detection threshold and not included in the final analysis (*n* = 14). CRP = C-reactive protein; CK = creatine kinase; SEM = standard error of the mean.

To assess whether the proposed exercise paradigm induced muscle damage, we analyzed log transformed serum levels of CK, CRP, and myoglobin. The changes in CRP and myoglobin were not statistically elevated at any point during the exercise paradigm, compared to baseline levels (**Fig. 6**). Serum CK concentrations for the population decreased below baseline during both walking tasks. (Flat: *P* = 0.05; Tilt: *P* = 0.003) but increased above baseline 3 percentage 1 hour post exercise (*P* = 0.026). This increase post exercise was largely due to the response of only 1 out of the 14 subjects analyzed (+18%). When the outlier CK value was not included in the statistical analysis, the population response at 1 hour rest post exercise compared to baseline was no longer significant. (*P* = 0.075) It is worth noting that the subject’s CK value at baseline (71 U/L) was within the reported population range (see descriptive population statistics in Suppl. Table S2), and the induced change in the other biomarkers for this subject were within the observed population response. As a result, it is difficult to determine the underlying factors responsible for the deviated CK change in this singular subject.

## Discussion

In this study, we incorporated a cross-tilting feature to a short-term walking exercise protocol to delineate the effects of abnormal joint mechanical loading on the kinetic behavior of inflammatory and degradative biomolecules. The present circumstantial data collected in young, healthy men showed that when mechanical loading was applied to the lower body joints during a cross-tilted walking task, blood serum concentrations of COMP, as well as pro-inflammatory (TNF-α and IL-1β) and anti-inflammatory (IL-10 and TGF-β) biomarkers yielded acute synchronous increases, while increases in collagenase II (MMP-13) was delayed. Our biomarker results also showed that serum COMP concentrations returned to basal levels after an hour of rest post exercise, while TNF-α, IL-1β, and IL-10 concentrations remained elevated. Collectively, the serum biomarker data generated from the present study supported our hypothesis that the increased expression of pro- and anti-inflammatory cytokines and degradative *metalloproteinases* would be positively associated with the expression of COMP during the exercise paradigm. This study also builds upon prior research^4,25,26^ investigating the prognostic potential of DAMPs such as COMP as biomarkers related to joint cartilage health. Based on our findings in this exercise study, we argue that the stress-induced expression of COMP in serum is likely associated with the onset of acute low yield inflammation and cartilage tissue remodeling.

While COMP is present in other connective tissues like tendon and synovium, evidence in favor of serum COMP being primarily derived from articular cartilage is compelling. For example, at the knee, COMP concentrations were 6-fold higher than the joint tendons.^7^ This skewed density of COMP in cartilage, supports the likelihood that exercise induced change in this study is due to stress to the articular cartilage. Indeed, these assumptions have been employed by other research groups exploring exercise and COMP relationship in joint health.^27,28^ Furthermore, serum COMP has also been utilized as a reliable prognostic marker for degenerative diseases that impact articular cartilage health, like osteoarthritis (OA) and rheumatoid arthritis (RA).^26^ Taken together, these reports support the notion that an assessment of the serum concentrations of COMP during exercise can be used as a metric of stress-induced cartilage breakdown in the joint.

COMP kinetics have been previously investigated as the main outcome during flat walking exercise.^29,30^ After the completion of a flat walking task, serum COMP concentrations in human subjects transiently increased and then rapidly decreased within a 2-hour timespan. Moreover, serum COMP levels in long distance athletes have been reported to increase over 4-fold from baseline and remain elevated for up to 48 hours after the completion of a marathon^15^ or an ultramarathon.^31^ The combined findings from these previous studies suggest that COMP diffusion patterns are sensitive to the magnitude and frequency of mechanical loading on lower limb joints. While the results of the present study demonstrate significant associations between an increase in joint loading and serum COMP expression during exercise compared to resting baseline, we did not detect significant increases of serum COMP as joint loading increased between flat and tilted walking (*P* = 0.874). It remains to be seen if varying the relative duration of exercise (flat-tilt) would yield significant difference in COMP expression. Indeed, one could imagine a modification to the existing paradigm that enables further experimentation with the relative impact of the duration of mechanical stimulus on stress-induced cartilage breakdown.

Accumulating evidence has shown that the human synovium exhibits a complex network of vasculature and lymphatic vessels that contribute to the maintenance of joint metabolic homeostasis.^32,33^ Indeed, the synovial lymphatic system (SLS) has been implicated in clearing molecular structures within the joint, including DAMPs and pro-inflammatory cytokines, at multiple time scales with respect to molecular mass.^33^ Specifically, previous groups have measured the rate of uptake of fluorescently tagged dextran or poly-ethylene glycol (PEG) molecules (comparable in mass compared to cytokines: 10-40 kDa) from rat joint cavities by the SLS (half-life = 3.26 h).^34,35^ Note that while the half-life of these molecules is in the order of 3 hours, it is clear that significant lymphatic uptake of the biomarkers occurred throughout the duration of the present exercise task from the joint. This evidence of plausible short-term lymphatic uptake of molecules from the synovium supports our fundamental assumption that the observed serum levels of biomarkers over the short term in this study originated from the joint.

Positive correlations between the expressions of COMP and the pro-inflammatory cytokine TNF-α were found throughout and post exercise (see **Fig. 5**). Although a direct relationship between COMP and pro-inflammatory cytokine expression has been suggested in past strenuous exercise studies^15,36^ and cartilage disease studies,^8^ experimental evidence has not supported the existence of this relationship in joint specific cells.^37,38^ Alternatively, other known DAMPs associated with cartilage damage such as fibronectin fragments (FnFs) have been shown to trigger pro-inflammatory responses in joint cells. FnFs have been shown to bind and activate toll-like receptors (TLRs), which are present in the cellular membranes of chondrocytes and synovial fibroblasts.^39^ Stimulation of these TLRs initiates activity of all 3 MAPK pathways (i.e. ERK1/2, c-Jun N-terminal kinase, and p38α), ultimately upregulating NF-kB, which results in the production of inflammatory cytokines such as TNF-α and catabolic MMPs.^39,40^ While it was not possible to measure FnFs quantitatively using the present methodology, previous biomarker studies have shown that COMP and FnF release in synovial fluid are both directly related to articular cartilage degradation and OA progression.^39,41^ Thus, future clinical studies should utilize an expanded biochemical analysis on DAMPs such as FnF and other clinically validated indicators of cartilage remodeling such as C-terminal telopeptide of collagen type II (CTX-II) to quantify the impact of acute loading on articular cartilage.^42^

Interestingly, our results also showed that COMP was positively correlated to the expression of the anti-inflammatory cytokine IL-10 during and after exercise. Most previous studies on IL-10 have been conducted in animal or *ex vivo* model systems and only a few investigations have measured stress-mediated changes of IL-10 in humans.^43,44^ To our knowledge, the *in vivo* concentration response of IL-10 to stress-mediated releases of DAMPs such as COMP has not been thoroughly investigated. IL-10 has been found to display chondroprotective properties by antagonizing important steps in the suggested onset of low-grade inflammation, such as suppressing the release of inflammatory cytokines by synoviocytes and chondrocytes,^45^ and stimulating the polarization of macrophages into an anti-inflammatory phenotype (M2c).^46^ Our serum data also indicated a unique elevation of the regenerative growth factor TGF-β throughout exercise which likely targets the stress-mediated loss in COMP from articular cartilage.^24^ While it is tempting to assume that the synchronous changes of these pro and anti-inflammatory mediators are driven primarily by the abnormal mechanical stimulus introduced in the present construct, future experimental work is needed to properly delineate the specific mechanosensitive pathways driving the present biological response.

From our panel of degradative biomarkers, only serum concentrations of the collagenases MMP-1 and MMP-13 yielded changes during exercise. MMP-13 expression was also significantly correlated to COMP throughout and post exercise; however, statistically significant increases from baseline were achieved only after 30 minutes of tilted walking, not flat walking, highlighting a delayed kinetic response. The reason for this delayed response is currently unclear; however as indicated earlier in the discussion, the mass related clearance rate from the joint through the SLS may contribute to the observed delayed response in these molecules.

To date, the magnitude of biomarker response studies that use normal flat walking as the biomechanical stimulus has been relatively low.^27,47^ While other exercise studies have shown much larger biomarker concentration changes in response to using high intensity or long distance running as the biomechanical stimulus, the overall significance as it relates to joint cartilage is limited. The main issue is that high-impact, long-duration exercise can introduce a number of confounding factors such as muscle damage which leads to the onset of a systemic inflammatory response.^48^ Indeed, even for the short duration flat walking exercise studies alluded to earlier,^27,30^ muscle damage biomarkers were not reported. Exercise induced muscle damage is generally characterized by the leakage of muscle proteins into circulation, such as CRP, CK, and myoglobin.^49^ In our examination, we failed to detect any statistical change in serum CRP and myoglobin concentrations in the population, or in serum CK concentrations reported in 13 participants (excluding the outlier described in Results). Furthermore, a comparable exercise study in young men by Pilat and colleagues reported the serum CK concentration change from resting baseline to be 30 percentage 1 hour post strenuous exercise, a percent change that is substantially higher in comparison to even the outlier value of the one singular subject reported in this study (+18%).^50^ Moreover, adding CRP and myoglobin as secondary metrics of exercise induced muscle damage is significant in the context of the observed decreasing CK values at the end of flat and tilted walking compared to baseline, a counter intuitive result (see **Fig. 6**). Thus, it is unlikely that the exercise employed herein induced any muscle damage that would have confounded our present results.

It is also important to note that the expression of IL-6 was not detected in blood serum throughout the duration of the exercise paradigm. IL-6 is a well-known pleiotropic cytokine produced locally by different cell types and is also the founding member of the myokine family of muscle produced cytokines.^9,51,52^ Previous strenuous exercise studies have shown noticeable systemic increases in serum IL-6 levels after muscle damage.^52^ In this study, IL-6 was measured using a conventional Thermo Fischer Luminex multiplex assay system and concentrations of the protein did not exceed the assay’s lower threshold of detection (10.18 pg/ml) in any of the collected samples. IL-6 has been successfully measured in other human studies; however, groups have employed specialized ultrasensitive kits to measure this biomarker in serum.^53^ Hence, it is difficult at present to provide IL-6 data as a secondary confirmation of inflammation caused by muscle damage.

The observed kinetic behavior for our biomarker panel throughout and after this short-term testing paradigm, establishes the potential viability of this construct in a clinical context. For instance, the current paradigm can be employed over the short duration of a single lab visit to assess the recovery progress of patients who suffered from a joint injury event, while also serving as a potential testing platform to determine the efficacy of acute biologic and pharmacological intervention on patients who suffer from chronic diseases such as OA. By revealing the kinetic details of the biological indicators driving acute low-grade joint inflammation and cartilage remodeling, it will allow clinicians and researchers to achieve deeper insight into the joint physiology in a healthy or diseased state.

### Limitations

It can be argued that this study design lacks an adequate controlled data set to determine whether the present biomarker changes are a result of increased joint stress during the cross-tilted treadmill walking stage or from accumulated tissue injury during the flat walking stage. Indeed, while the primary goal of the study was to determine within subject change of serum biomarker levels at baseline and during increased joint stress exposure, an additional session of flat walking over the same exercise duration of 1 hour, in the same subject cohort, in a separate visit is required to make this comparison. Our lack of inclusion of this control session was primarily attributed to recruitment barriers and the overhead cost of performing proteomics on the collection of blood samples over 2 visits.

Another limitation of the experimental design involves the confounding effect of increased venous blood flow from exercise on the measurement of biomarkers in blood serum. This exercise induced surge in venous flow rate likely increases the presence of circulatory protein concentrations in blood samples collected during exercise. Future analysis that incorporates a simultaneous recording of systemic and local blood flow paired with sample total protein analysis will allow for the assessment of this potential confounder.

One could also argue that differences in testosterone levels across participants may influence the expression of the inflammatory biomarkers. Previous groups have reported the potential anti-inflammatory effects of testosterone on synovial metabolism^54^; thus, it is important to minimize the potential confounding effects of daily circadian variations of testosterone levels in male participants through precise scheduling.^55^

## Conclusion

In conclusion, stress-mediated increases of COMP correlated with changes in both pro- and anti-inflammatory biomarkers simultaneously, while changes in biomarkers involved with articular cartilage remodeling where delayed. Although this study focused on healthy human controls, the current format can be employed to assess existing or future biologic (PRP)^56^ and/or pharmacological (cortisol injection)^57^ treatments targeting specific mechanosensitive molecular mechanisms and pathways associated with low-grade joint inflammation and likely cartilage breakdown. Furthermore, the exercise construct presented herein is very appealing in the sense that modifications to the magnitude and duration of the constituencies of the walking stimulus provide the possibility for future investigations involving dose response analysis with and without interventions.

## Supplemental Material

sj-docx-1-car-10.1177_19476035231193089 – Supplemental material for Low-Grade Inflammatory Mediators and Metalloproteinases Yield Synchronous and Delayed Responses to Mechanical Joint Loading Supplemental material, sj-docx-1-car-10.1177_19476035231193089 for Low-Grade Inflammatory Mediators and Metalloproteinases Yield Synchronous and Delayed Responses to Mechanical Joint Loading by Conner W. Hutcherson, Michelle Mao, Bhaskar Thakur and Yasin Y. Dhaher in CARTILAGE

sj-docx-2-car-10.1177_19476035231193089 – Supplemental material for Low-Grade Inflammatory Mediators and Metalloproteinases Yield Synchronous and Delayed Responses to Mechanical Joint Loading Supplemental material, sj-docx-2-car-10.1177_19476035231193089 for Low-Grade Inflammatory Mediators and Metalloproteinases Yield Synchronous and Delayed Responses to Mechanical Joint Loading by Conner W. Hutcherson, Michelle Mao, Bhaskar Thakur and Yasin Y. Dhaher in CARTILAGE

sj-docx-3-car-10.1177_19476035231193089 – Supplemental material for Low-Grade Inflammatory Mediators and Metalloproteinases Yield Synchronous and Delayed Responses to Mechanical Joint Loading Supplemental material, sj-docx-3-car-10.1177_19476035231193089 for Low-Grade Inflammatory Mediators and Metalloproteinases Yield Synchronous and Delayed Responses to Mechanical Joint Loading by Conner W. Hutcherson, Michelle Mao, Bhaskar Thakur and Yasin Y. Dhaher in CARTILAGE
