# Supplementary material for: Low-Grade Inflammatory Mediators and Metalloproteinases Yield Synchronous and Delayed Responses to Mechanical Joint Loading
Source: Cartilage. 2023 Aug 24;15(4):417–27. doi: 10.1177/19476035231193089 (PMC11526223; doi:10.1177/19476035231193089)
Supplement: sj-docx-1-car-10.1177_19476035231193089 – Supplemental material for Low-Grade Inflammatory Mediators and Metalloproteinases Yield Synchronous and Delayed Responses to Mechanical Joint Loading [file sj-docx-1-car-10.1177_19476035231193089.docx]

| Outcome | Time | Beta Coefficient | 95% CI LL | 95% CI UL | P Value |
| --- | --- | --- | --- | --- | --- |
| log COMP | Baseline vs Flat | 0.332 | 0.217 | 0.446 | <0.001 |
|  | Baseline vs Tilt | 0.346 | 0.232 | 0.460 | <0.001 |
|  | Baseline vs Rest | -0.002 | -0.114 | 0.110 | 0.980 |
| log TNF-α | Baseline vs Flat | 0.224 | 0.080 | 0.368 | 0.002 |
|  | Baseline vs Tilt | 0.359 | 0.214 | 0.503 | <0.001 |
|  | Baseline vs Rest | 0.219 | 0.078 | 0.361 | 0.002 |
| log IL-1β | Baseline vs Flat | 0.236 | 0.082 | 0.390 | 0.003 |
|  | Baseline vs Tilt | 0.283 | 0.130 | 0.436 | <0.001 |
|  | Baseline vs Rest | 0.148 | -0.003 | 0.298 | 0.050 |
| log IL-10 | Baseline vs Flat | 0.214 | 0.080 | 0.348 | 0.002 |
|  | Baseline vs Tilt | 0.326 | 0.193 | 0.460 | <0.001 |
|  | Baseline vs Rest | 0.210 | 0.079 | 0.341 | 0.002 |
| log TGF-β | Baseline vs Flat | 0.186 | 0.039 | 0.334 | 0.013 |
|  | Baseline vs Tilt | 0.283 | 0.136 | 0.430 | <0.001 |
|  | Baseline vs Rest | 0.101 | -0.043 | 0.246 | 0.170 |
| log MMP-1 | Baseline vs Flat | 0.204 | 0.067 | 0.341 | 0.003 |
|  | Baseline vs Tilt | 0.174 | 0.041 | 0.307 | 0.010 |
|  | Baseline vs Rest | 0.023 | -0.106 | 0.153 | 0.720 |
| log MMP-9 | Baseline vs Flat | 0.086 | -0.134 | 0.306 | 0.440 |
|  | Baseline vs Tilt | 0.017 | -0.206 | 0.240 | 0.880 |
|  | Baseline vs Rest | -0.028 | -0.240 | 0.185 | 0.800 |
| log TIMP-1 | Baseline vs Flat | 0.009 | -0.039 | 0.057 | 0.710 |
|  | Baseline vs Tilt | 0.038 | -0.010 | 0.085 | 0.120 |
|  | Baseline vs Rest | -0.031 | -0.078 | 0.016 | 0.190 |
| log MMP-13 | Baseline vs Flat | 0.052 | -0.074 | 0.179 | 0.420 |
|  | Baseline vs Tilt | 0.198 | 0.071 | 0.325 | 0.002 |
|  | Baseline vs Rest | 0.038 | -0.085 | 0.161 | 0.540 |
| log CRP | Baseline vs Flat | -0.004 | -0.117 | 0.110 | 0.950 |
|  | Baseline vs Tilt | -0.041 | -0.154 | 0.072 | 0.480 |
|  | Baseline vs Rest | -0.044 | -0.155 | 0.067 | 0.440 |
| log CK | Baseline vs Flat | 0.037 | 0.000 | 0.073 | 0.050 |
|  | Baseline vs Tilt | 0.055 | 0.019 | 0.092 | 0.003 |
|  | Baseline vs Rest | 0.041 | 0.005 | 0.077 | 0.026* |
| log Myoglobin | Baseline vs Flat | -0.007 | -0.034 | 0.020 | 0.620 |
|  | Baseline vs Tilt | -0.005 | -0.032 | 0.023 | 0.750 |
|  | Baseline vs Rest | -0.011 | -0.038 | 0.016 | 0.420 |

* includes 14 subjects with one outlier displaying +18% change from baseline. See more details in Results.

Table S1: Mixed Effects Model Results
