# Supplementary material for: Low-Grade Inflammatory Mediators and Metalloproteinases Yield Synchronous and Delayed Responses to Mechanical Joint Loading
Source: Cartilage. 2023 Aug 24;15(4):417–27. doi: 10.1177/19476035231193089 (PMC11526223; doi:10.1177/19476035231193089)
Supplement: sj-docx-2-car-10.1177_19476035231193089 – Supplemental material for Low-Grade Inflammatory Mediators and Metalloproteinases Yield Synchronous and Delayed Responses to Mechanical Joint Loading [file sj-docx-2-car-10.1177_19476035231193089.docx]

|  |  | COMP (pg/ml) | TNF-α (pg/ml) | IL-1β (pg/ml) | IL-10 (pg/ml) | TGF-β (pg/ml) | MMP-1 (pg/ml) | MMP-9 (pg/ml) | TIMP-1 (pg/ml) | MMP-13 (pg/ml) | CRP  (mg/L) | CK (U/L) | Myoglobin (ng/ml) | |
| --- | --- | --- | --- | --- | --- | --- | --- | --- | --- | --- | --- | --- | --- | --- |
|  | Baseline |  |  |  |  |  |  |  |  |  |  |  | |  |
| N |  | 15 | 15 | 15 | 15 | 15 | 15 | 15 | 15 | 11 | 14 | 14 | | 15 |
| Mean |  | 28998.76 | 151.30 | 92.06 | 39.42 | 69068.98 | 104.61 | 13606.66 | 238200.20 | 389.05 | 5.26 | 178.00 | | 4.92 |
| SD |  | 28033.81 | 142.65 | 58.25 | 32.03 | 45467.73 | 186.25 | 7997.61 | 141297.00 | 304.29 | 0.85 | 145.65 | | 0.79 |
| Median |  | 13241.10 | 99.22 | 83.06 | 27.85 | 55684.79 | 27.34 | 12705.82 | 152960.00 | 397.26 | 5.01 | 119.00 | | 4.72 |
| 1st quartile |  | 6837.75 | 50.84 | 45.03 | 12.12 | 42002.88 | 18.36 | 8526.77 | 117178.20 | 83.93 | 4.80 | 71.00 | | 4.52 |
| 3rd quartile |  | 44383.75 | 192.94 | 130.14 | 70.74 | 96580.23 | 97.74 | 14821.82 | 377213.20 | 612.45 | 5.40 | 229.00 | | 5.01 |
|  | Flat |  |  |  |  |  |  |  |  |  |  |  | |  |
| N |  | 14 | 14 | 14 | 14 | 14 | 13 | 13 | 14 | 10 | 14 | 13 | | 14 |
| Mean |  | 38897.33 | 195.48 | 120.88 | 49.69 | 87074.85 | 101.09 | 13158.47 | 227314.40 | 446.38 | 5.35 | 165.77 | | 4.90 |
| SD |  | 33914.46 | 176.20 | 66.26 | 37.20 | 46061.76 | 178.97 | 6283.96 | 138624.20 | 328.68 | 1.39 | 139.96 | | 0.38 |
| Median |  | 24821.45 | 114.41 | 106.27 | 31.79 | 83412.55 | 32.82 | 11845.21 | 139788.90 | 429.98 | 4.95 | 94.00 | | 4.84 |
| 1st quartile |  | 12458.68 | 89.55 | 90.51 | 27.82 | 56209.66 | 23.02 | 10046.67 | 126126.80 | 103.89 | 4.40 | 76.00 | | 4.63 |
| 3rd quartile |  | 52152.39 | 214.46 | 143.05 | 58.34 | 112708.80 | 63.23 | 14028.43 | 361125.10 | 666.33 | 5.60 | 166.00 | | 5.04 |
|  | Tilt |  |  |  |  |  |  |  |  |  |  |  | |  |
| N |  | 14 | 14 | 14 | 14 | 14 | 14 | 13 | 14 | 10 | 14 | 13 | | 14 |
| Mean |  | 41903.04 | 227.28 | 123.25 | 55.89 | 93006.86 | 123.10 | 15868.12 | 256501.10 | 504.68 | 5.08 | 161.62 | | 4.84 |
| SD |  | 36058.85 | 231.07 | 71.57 | 46.33 | 54608.82 | 192.40 | 14183.02 | 152197.10 | 356.34 | 0.67 | 120.82 | | 0.38 |
| Median |  | 29200.73 | 117.20 | 120.94 | 35.39 | 97713.38 | 35.20 | 12585.72 | 220604.70 | 540.49 | 4.85 | 93.00 | | 4.77 |
| 1st quartile |  | 12811.03 | 87.73 | 60.33 | 22.95 | 53904.32 | 22.88 | 9192.30 | 119803.00 | 93.11 | 4.70 | 78.00 | | 4.57 |
| 3rd quartile |  | 65670.72 | 283.18 | 158.50 | 78.99 | 120998.90 | 136.13 | 14922.58 | 397877.30 | 777.06 | 5.60 | 182.00 | | 5.05 |
|  | Rest |  |  |  |  |  |  |  |  |  |  |  | |  |
| N |  | 15 | 15 | 15 | 15 | 15 | 15 | 15 | 15 | 11 | 15 | 14 | | 15 |
| Mean |  | 25048.00 | 164.10 | 101.99 | 43.99 | 72302.51 | 99.37 | 14573.29 | 231778.60 | 383.07 | 5.13 | 183.14 | | 4.84 |
| SD |  | 18966.52 | 118.57 | 56.85 | 28.50 | 37087.27 | 163.55 | 10960.51 | 139209.20 | 286.98 | 1.21 | 144.94 | | 0.42 |
| Median |  | 12865.22 | 118.04 | 91.83 | 38.35 | 74559.87 | 24.18 | 12171.22 | 146341.70 | 398.88 | 4.60 | 127.50 | | 4.79 |
| 1st quartile |  | 9475.32 | 79.71 | 68.05 | 23.63 | 47683.71 | 21.80 | 7662.70 | 107400.90 | 89.33 | 4.40 | 82.00 | | 4.57 |
| 3rd quartile |  | 41049.81 | 221.75 | 123.33 | 76.41 | 97364.88 | 120.80 | 15306.92 | 377628.80 | 645.24 | 5.30 | 244.00 | | 5.06 |
