# Supplementary material for: Low-Grade Inflammatory Mediators and Metalloproteinases Yield Synchronous and Delayed Responses to Mechanical Joint Loading
Source: Cartilage. 2023 Aug 24;15(4):417–27. doi: 10.1177/19476035231193089 (PMC11526223; doi:10.1177/19476035231193089)
Supplement: sj-docx-3-car-10.1177_19476035231193089 – Supplemental material for Low-Grade Inflammatory Mediators and Metalloproteinases Yield Synchronous and Delayed Responses to Mechanical Joint Loading [file sj-docx-3-car-10.1177_19476035231193089.docx]

|  |  | COMP | TNF-α | IL-1β | IL-10 | TGF-β | MMP-1 | MMP-9 | TIMP-1 | MMP-13 | CRP | CK | Myoglobin |
| --- | --- | --- | --- | --- | --- | --- | --- | --- | --- | --- | --- | --- | --- |
|  | Baseline |  |  |  |  |  |  |  |  |  |  |  |  |
| N |  | 15 | 15 | 15 | 15 | 15 | 15 | 15 | 15 | 11 | 14 | 14 | 15 |
| Mean |  | 9.84 | 4.63 | 4.34 | 3.34 | 10.92 | 3.76 | 9.39 | 12.20 | 5.54 | 1.65 | 4.91 | 1.58 |
| SD |  | 0.98 | 0.93 | 0.63 | 0.87 | 0.75 | 1.23 | 0.52 | 0.64 | 1.09 | 0.15 | 0.74 | 0.14 |
|  | Flat |  |  |  |  |  |  |  |  |  |  |  |  |
| N |  | 14 | 14 | 14 | 14 | 14 | 13 | 13 | 14 | 10 | 14 | 13 | 14 |
| Mean |  | 10.19 | 4.95 | 4.64 | 3.66 | 11.21 | 3.83 | 9.41 | 12.15 | 5.70 | 1.65 | 4.86 | 1.59 |
| SD |  | 0.91 | 0.85 | 0.61 | 0.75 | 0.67 | 1.12 | 0.39 | 0.62 | 1.10 | 0.22 | 0.70 | 0.07 |
|  | Tilt |  |  |  |  |  |  |  |  |  |  |  |  |
| N |  | 14 | 14 | 14 | 14 | 14 | 14 | 13 | 14 | 10 | 14 | 13 | 14 |
| Mean |  | 10.25 | 5.01 | 4.64 | 3.70 | 11.22 | 3.98 | 9.41 | 12.26 | 5.84 | 1.62 | 4.86 | 1.57 |
| SD |  | 0.95 | 0.96 | 0.64 | 0.86 | 0.78 | 1.24 | 0.73 | 0.67 | 1.06 | 0.13 | 0.67 | 0.08 |
|  | Rest |  |  |  |  |  |  |  |  |  |  |  |  |
| N |  | 15 | 15 | 15 | 15 | 15 | 15 | 15 | 15 | 11 | 15 | 14 | 15 |
| Mean |  | 9.83 | 4.85 | 4.48 | 3.56 | 11.00 | 3.80 | 9.36 | 12.17 | 5.57 | 1.61 | 4.95 | 1.57 |
| SD |  | 0.81 | 0.77 | 0.58 | 0.73 | 0.71 | 1.15 | 0.69 | 0.64 | 1.01 | 0.21 | 0.73 | 0.08 |

Table S3: Log Serum Biomarker Concentrations
